# Surrogate endpoints in diabetic kidney disease: current perspectives and future directions

**DOI:** 10.3389/fendo.2025.1557813

**Published:** 2025-05-21

**Authors:** Can Cao, Jiale Zhang, Tong Ma, Juan Miao, Weiwei Sun

**Affiliations:** Department of Nephrology and Endocrinology, Dongzhimen Hospital, Beijing University of Chinese Medicine, Beijing, China

**Keywords:** diabetic kidney disease, surrogate endpoints, ESRD, eGFR slop, UACR, novel biomarkers

## Abstract

Diabetic kidney disease (DKD) represents a leading complication of diabetes, frequently progressing to end-stage renal disease (ESRD), which significantly impairs patients’ quality of life and imposes substantial healthcare burdens. Consequently, early detection and intervention in DKD are paramount. The incorporation of surrogate endpoints in clinical trials has emerged as a pivotal strategy for assessing the efficacy of novel therapies, facilitating the reduction of trial duration and associated costs. Currently, the rate of change in estimated glomerular filtration rate (eGFR) and urinary albumin excretion, either independently or in combination, serve as reliable surrogate endpoints for evaluating DKD progression. Although novel biomarkers such as KIM-1 and TNFR2 are not yet recommended as standalone surrogate endpoints for DKD, they hold potential when used in combination with established markers, such as eGFR slope and urinary albumin change rate, to improve the prediction of ESRD risk. While omics-based indicators demonstrate promise in DKD research, their utility requires further validation, particularly through long-term follow-up and dynamic monitoring, to establish their effectiveness and clinical applicability. Future research should prioritize the validation and optimization of potential surrogate endpoints through long-term follow-up studies and large-scale cohorts.

## 1 Introduction

In individuals with type 2 diabetes, nearly 50% will progress to diabetic kidney disease (DKD) (1). DKD is the leading cause of end-stage renal disease (ESRD), necessitating renal replacement therapy (2, 3). Moreover, it is a major contributor to cardiovascular morbidity and overall mortality in diabetic patients (4, 5). As the disease progresses, the financial burden of managing DKD escalates, and by the time patients reach the uremic stage, the economic strain on families becomes profound. This not only diminishes patients’ quality of life but also imposes a substantial societal burden, placing significant pressure on national healthcare resources (6). Consequently, the early detection and intervention in DKD are of paramount importance.

Regulatory bodies, such as the U.S. Food and Drug Administration (FDA), require that the benefits of new pharmacological interventions must clearly outweigh potential risks. To meet this criterion, novel therapeutics must demonstrate substantial efficacy on clinically meaningful endpoints in rigorously conducted clinical trials, with therapeutic effects significantly surpassing any adverse reactions experienced by patients. This process necessitates considerable financial and human resource investment (7), compounded by the high dropout rates in late-stage trials, which further complicate the research landscape. As a result, there is a pressing need to identify and validate surrogate endpoints to assess the efficacy and safety of new drugs, which could potentially substitute for traditional clinically endpoints. The advantages of employing surrogate endpoints in clinical trials include the potential for earlier occurrence and easier assessment, thus reducing trial duration and associated costs (8). Indeed, surrogate endpoints have become integral to the drug approval process. Between 2005 and 2012, nearly half of the approved drugs relied primarily on surrogate endpoints as key measures in clinical trials (9). However, the validity and utility of biomarkers as surrogate endpoints require rigorous validation. Non-compliant candidate indicators could result in an overestimation of therapeutic benefits or delays in the introduction of effective treatments. Although no standardized criteria exist for surrogate endpoints, they should ideally be measurable, occur earlier than clinical endpoints, and be supported by robust evidence linking them to the clinical outcomes of interest. In clinical intervention trials, results derived from surrogate endpoints should align with those from clinical endpoints (8).

In clinical trials targeting the progression of diabetic kidney disease (DKD), end-stage renal disease (ESRD) is typically considered a clinically endpoint. However, the progression to ESRD may span several years, necessitating extensive and complex clinical trials to assess drug efficacy. For patients with rapidly progressing or advanced DKD, achieving sufficient follow-up time for endpoint events is often challenging, while early-stage DKD patients require prolonged follow-up, which imposes substantial demands on financial and human resources. Despite these challenges, early intervention in DKD has been shown to be more effective in delaying disease progression. Consequently, there is an urgent need for surrogate endpoints to evaluate the efficacy of new therapeutics in clinical trials, particularly for those with early-stage DKD. Surrogate endpoints would help shorten follow-up periods, improve patient compliance, and reduce costs, thereby accelerating clinical trials and drug development for DKD (10). This article seeks to review the current landscape of surrogate endpoints in diabetic kidney disease and explore their future potential.

## 2 Research on glomerular filtration rate and its rate of change

Glomerular filtration refers to the process by which blood is filtered through the glomerular capillaries into the Bowman’s capsule. The volume of ultrafiltrate produced by the kidneys per unit of time is termed the glomerular filtration rate (GFR), which reflects the filtering capacity of the glomeruli and varies in response to physiological and clinical factors. In animal models of kidney disease, GFR often initially increases due to hyperfiltration and glomerular hypertrophy. However, as the disease progresses, GFR gradually declines. A decline in GFR over a specified period is considered a key indicator of renal function deterioration, with end-stage renal disease (ESRD) defined by a GFR < 15 ml/min/1.73 m². Although GFR is the gold standard for assessing renal function, it cannot be directly measured in humans. Instead, the estimated glomerular filtration rate (eGFR) is calculated using serum levels of endogenous filtration markers, such as creatinine and cystatin C (11). Due to the ease of measurement, eGFR is widely utilized in clinical practice to monitor renal function progression. While direct measurement of GFR is not practical for everyday practice, it is utilized in research studies using inulin, I-iothalamate or iohexol (12). Given that eGFR and its rate of change represent critical pathways in the progression of diabetic kidney disease (DKD) to ESRD, this area remains a major focus of ongoing research.

### 2.1 Rate of decline in glomerular filtration rate

The decline in estimated glomerular filtration rate (eGFR) is a pivotal process in the progression of diabetic kidney disease (DKD) to end-stage renal disease (ESRD), with its dynamic changes serving as a predictor of endpoint events. As such, eGFR decline has emerged as a surrogate endpoint for assessing disease progression. In recent years, significant attention has been directed toward identifying the optimal threshold for eGFR decline to serve as a surrogate endpoint, with ongoing debates regarding whether a 30% or 40% decline should be used. This issue has spurred considerable research both domestically and internationally, leading to important advancements in the field.

Initially, serum creatinine doubling was considered a surrogate endpoint for diabetic kidney disease (DKD), with a corresponding 57% decline in estimated glomerular filtration rate (eGFR) when calculated using the CKD-EPI equation. A systematic meta-analysis by Jun (13), which included over 20 randomized controlled trials, demonstrated that the effect size of serum creatinine doubling in relation to ESRD is close to 1, suggesting its potential as a reliable surrogate endpoint for ESRD. Serum creatinine doubling, equivalent to a 50% reduction in eGFR, reflects a substantial loss of renal function and is a strong predictor of progression to renal failure, thus facilitating shorter follow-up periods in clinical trials. However, serum creatinine doubling is predominantly applicable to late-stage events in DKD, which limits its utility in significantly reducing sample sizes or trial durations. As a result, there has been growing support for lowering the threshold for eGFR decline, with proposals to use a 30% or 40% reduction as a surrogate endpoint for DKD. A smaller decline in eGFR may result in a higher incidence of surrogate endpoints events, particularly in patients with normal or mildly reduced eGFR. In an analysis of two randomized controlled trials, Lambers Heerspink (14) observed that in the RENAAL study, event rates for 40% and 30% eGFR declines increased from 33.6% to 48.8% and 61.4%, respectively; in the IDNT trial, rates rose from 28.1% to 39.1% and 51.5%. These findings suggest that smaller declines in eGFR may precipitate earlier surrogate endpoints events. In addition to occurring earlier, the validity of smaller eGFR declines as surrogate endpoints must be further validated, particularly regarding their alignment with the occurrence of ESRD events.

Coresh et al. (15) conducted a meta-analysis encompassing over 1.7 million patients with kidney disease worldwide, examining the relationship between percentage changes in eGFR over a two-year period and the risks of ESRD and all-cause mortality, adjusting for confounding factors and baseline eGFR. The results revealed that declines of 57% and 30% in eGFR were associated with adjusted hazard ratios for ESRD of 32.1 (95% CI 22.3–46.3) and 5.4 (95% CI 4.5–6.4), respectively. 57% decline in eGFR was linked to more than a 30-fold increase in the risk of ESRD, establishing it as a robust surrogate endpoint. Similarly, 30% reduction in eGFR was associated with over a fivefold increased risk of ESRD, suggesting its potential as a surrogate endpoint for eGFR changes over two years. Additionally, Lambers Heerspink et al. (16) performed a meta-analysis of 37 randomized controlled trials, finding that a 30% decline in eGFR, irrespective of baseline eGFR, proteinuria, or interventions, was associated with a hazard ratio of 9.6 (95% CI 7.3–12.6) for endpoint events (eGFR < 15 ml/min/1.73 m² or doubling of serum creatinine) after two years of follow-up. This strong association further supports the use of a 30% decline in eGFR as a surrogate endpoint in clinical trials. Taken together, these findings suggest that surrogate endpoints closely correlated with ESRD may be viable, with a 30% decline in eGFR within 2 to 3 years serving as a potential surrogate endpoint for DKD, provided that acute influences on eGFR are excluded (17). In the early stages of DKD, the rate of decline in eGFR is minimal, therefore, its use is not recommended.

### 2.2 Slope of glomerular filtration rate

As previously noted, a 30% decline in estimated glomerular filtration rate (eGFR) has been proposed as a surrogate endpoint; however, its applicability may be limited in certain populations, such as those with early-stage diabetic kidney disease (DKD), short follow-up durations for interventions, or in cases where medications exert acute effects on DKD. As kidney disease advances, the progressive and irreversible nature of DKD becomes more evident, underscoring the importance of early intervention to improve patient outcomes. Consequently, there is a need to explore novel surrogate endpoints for assessing the efficacy of interventions, particularly in the early stages of DKD. In 2018, the National Kidney Foundation (NKF), the U.S. Food and Drug Administration (FDA), and the European Medicines Agency (EMA) convened a scientific workshop to assess the feasibility of using the eGFR slope as a surrogate endpoint in early-stage kidney disease. Through meta-analyses of observational cohort studies, clinical trials, and simulation models, it was determined that the eGFR slope can serve as a reliable surrogate endpoint in clinical trials, provided that treatments do not induce acute effects on eGFR in the early stages of the disease (18).

The estimated glomerular filtration rate (eGFR) slope (19) can be classified into chronic and acute slopes. The acute slope refers to the average change in eGFR over a short follow-up period, typically spanning three months, while the chronic slope reflects the average change over a longer duration, typically exceeding one year. The total slope generally represents the difference in eGFR from baseline measurements to the end of the follow-up period. Grams (20) conducted a cohort meta-analysis involving millions of patients with kidney disease, with an average follow-up of 4.2 years, and found a significant association between the eGFR slope and the risk of ESRD. Specifically, within two years, patients exhibiting a decline in eGFR slope of less than 0.75 ml/min/1.73 m² demonstrated substantially reduced risks of ESRD, with adjusted hazard ratios (HR) of 0.70 for eGFR ≥ 60 ml/min/1.73 m² and 0.71 for eGFR < 60 ml/min/1.73 m², with no significant differences observed between the two groups. These findings support the consideration of eGFR slope as a viable surrogate endpoint for early DKD. Additionally, Greene (19) simulated eGFR slopes using data from 47 randomized controlled trials, revealing that the use of eGFR slope as a surrogate endpoint could shorten follow-up periods from 4–6 years to just 2 years, while reducing sample sizes by 14% to 39%.

Overall, the application of the eGFR slope as a surrogate endpoint can substantially reduce both the required sample size and follow-up duration, particularly in patients with early-stage DKD. When compared to using a 30% or 40% decline in eGFR as an endpoint, the eGFR slope offers the advantage of further shortening the follow-up period. However, it is important to recognize that if a drug induces acute effects on eGFR, extending the follow-up period may be necessary to account for the impact of these acute factors on eGFR measurements. The table below summarizes the advantages and limitations associated with using various thresholds of eGFR decline, as well as the eGFR slope, as surrogate endpoints (**Table 1**).

**Table 1:** Advantages and Limitations of eGFR as a Surrogate Endpoint in DKD.

Surrogate Endpoint	Advantages	Limitations
Doubling of Serum Creatinine (eGFR decline by 57%)	- Strong association with progression to end-stage renal disease (ESRD). - Reliable for late-stage DKD. - Widely recognized and established.	- Primarily applicable to late-stage DKD. - Limited use in early-stage disease. - Requires longer follow-up.
40% eGFR Decline within 2–3 Years	- Predictive of significant renal function deterioration. - More sensitive compared to serum creatinine doubling. - Applicable across stages.	- May not be as sensitive in early-stage DKD. - The threshold may need further validation. - Risk of including acute eGFR fluctuations.
30% eGFR Decline within 2–3 Years	- Identified as a reliable predictor of ESRD. - Shows early detection of progression. - Associated with a strong hazard ratio for ESRD.	- Acute changes in eGFR may affect results. - Requires further validation in some populations. - May not fully account for acute factors.
eGFR Slope	- Can assess gradual changes over time. - Useful in early-stage DKD. - Reduces follow-up duration and sample size requirements in clinical trials.	- Requires consistent, long-term data. - Sensitive to acute changes in eGFR. - May need longer follow-up in cases with acute eGFR changes.

## 3 Research on UACR and its changes

In diabetic patients, prolonged hyperglycemia triggers the formation of advanced glycation end products, induces hypoxia, activates oxidative stress, and stimulates the renin-angiotensin-aldosterone system (RAAS), inflammation, and the release of fibrotic mediators. These processes collectively contribute to endothelial and epithelial cell damage, leading to mesangial cell expansion, podocyte injury, and glomerular hypertrophy, which ultimately result in the development of UACR (21, 22). In diabetic kidney disease, an increase in UACR often precedes a decline in glomerular filtration rate (GFR). While the gold standard for assessing albuminuria is the 24-hour urine albumin excretion rate, its collection is cumbersome and impractical for routine clinical use. Surrogate endpoints, the urine albumin-to-creatinine ratio (UACR), is more convenient, as it can be measured using a single morning urine sample. UACR has demonstrated strong correlations with the 24-hour urine albumin excretion rate, thereby establishing its validity as a reliable surrogate endpoint for UACR. Under normal conditions, the urine albumin excretion rate is <30 mg/d (24-hour collection) or the urine ACR is <30 mg/g (morning sample). However, in diabetic patients with kidney damage, microalbuminuria (stage A2), characterized by a urine albumin excretion rate of 30–300 mg/d or urine ACR of 30–300 mg/g, may emerge. In some cases, macroalbuminuria (stage A3) develops, defined by a urine albumin excretion rate >300 mg/d or urine ACR >300 mg/g (23).

UACR has long been considered a critical predictor of diabetic kidney disease (DKD) progression. A meta-analysis encompassing 21,688 patients from 13 cohort studies demonstrated that, after adjusting for confounding variables, a urine albumin-to-creatinine ratio (UACR) exceeding eight times the normal threshold was strongly associated with increased mortality, with a risk ratio of 1.4 (95% confidence interval: 1.27–1.55). Furthermore, elevated UACR was significantly correlated with the risk of progression to end-stage renal disease (ESRD), yielding a risk ratio of 3.04 (95% confidence interval: 2.27–4.08). This analysis also highlighted a direct association between the severity of urine albumin excretion and the advancement of DKD (24). In addition, numerous other studies have corroborated the role of increased urine albumin excretion as a reliable predictor of ESRD, cardiovascular events, and overall mortality (25).

Until recently, only a limited number of observational studies have explored the relationship between temporal changes in UACR and the progression of diabetic kidney disease (DKD), shifting the focus from single-time-point assessments to repeated measurements. A follow-up study of 4,570 DKD patients over three years found that individuals with a urine albumin-to-creatinine ratio (UACR) change of ≥30% had a significantly higher risk of adverse renal outcomes, such as serum creatinine doubling or the need for renal replacement therapy, compared to those with stable UACR and estimated glomerular filtration rate (eGFR) levels (26). In a separate cohort of 8,766 type 2 diabetes patients, changes in UACR over two years were positively correlated with major renal outcomes, suggesting that sustained increases in UACR could independently predict renal complications (27). These findings indicate that dynamic changes in UACR may serve as a valuable surrogate endpoint for renal outcomes in DKD patients. However, further investigation is required to establish the critical thresholds for UACR changes that would solidify its role as a reliable surrogate endpoint. A meta-analysis demonstrated that a 30% reduction in UACR over two years was associated with a relatively low risk of end-stage renal disease (ESRD), particularly in patients with UACR >300 mg/g. Moreover, even in the early stages of DKD, a 30% decrease in UACR within two years could reduce the absolute risk of ESRD by over 1% after ten years (28). Additionally, a study involving 91,319 patients revealed that a ≥30% change in UACR over three years significantly increased the risk of renal outcomes, including eGFR <30 mL/min/1.73 m². In a cohort study involving 91,319 patients, the hazard ratios for a ≥30% increase in UACR and ≥30% decrease in eGFR were 1.78 (95% CI, 1.59-1.98) and 7.53 (95% CI, 6.70-8.45), for the outcome of advanced CKD. Compared with stable values of both, the hazard ratio for the combination of an increase in UACR and a decrease in eGFR was 15.15 (95% CI, 12.43-18.46) for the outcome of advanced CKD. The combination of changes in UACR and eGFR predicted kidney outcomes better than either alone (29). In DKD stage G3 and beyond, the progressive decline in eGFR reduces the reliability of UACR as a surrogate endpoint, limiting its clinical utility in these stages. This sentence indicates that the application of eGFR and UACR as surrogate endpoints in clinical trials is summarized in **Table 2**.

**Table 2:** Summary of Surrogate Endpoint Applications in clinical trials.

Clinical Trials	Sample size	Follow-up time	Endpoint	Surrogate endpoints
Jun M (27)	8766	7.7	defined as requirement for chronic dialysis or kidney transplantation or renal death	Changes in UACR
Neuen BL (29)	91319	2.9	kidney failure, advanced CKD (sustained eGFR <30 mL/min/1.73 m^2^)	Changes in UACR, 30% eGFR Decline
Bakris GL (30)	5734	2.6	kidney failure, death from renal causes	40% eGFR Decline
Perkovic V (31)	4401	2.62	ESRD, death from renal causes	Dubling of Serum Creatinine
Mosenzon O (32)	17160	4.2	ESRD	40% eGFR Decline
Gerstein HC (33)	9901	5.4	first occurrence of the composite endpoint of non-fatal myocardial infarction, non-fatal stroke, or death from cardiovascular causes	30% eGFR Decline, UACR
S Ruilope LM (34)	7473	3.4	kidney failure, renal death	57% eGFR Decline, Changes in albuminuria, eGFR slope, 40% eGFR Decline
Miyamoto S (35)	98	1	NA	Changes in albuminuria, eGFR slope
Mosenzon O (36)	17160	4.2	ESKD, renal death	eGFR slope, 30% eGFR Decline, 40% eGFR Decline, 57% eGFR Decline

In comparison to other surrogate endpoints linked to estimated glomerular filtration rate (eGFR), changes in the UACR occur more frequently, particularly during the early stages of DKD. UACR is a well-established risk factor for DKD progression and can serve as an effective surrogate endpoints, particularly in patients with elevated baseline UACR. In clinical trials, the impact of interventions on UACR can often be detected within a relatively short follow-up period, such as six months. When interventions fail to effectively reduce proteinuria levels, the combined assessment of UACR changes and eGFR slope may offer a robust surrogate endpoints for evaluating the efficacy of treatments in DKD. The table below summarizes the advantages and limitations of UACR as a surrogate endpoint (**Table 3**).

**Table 3:** Advantages and Limitations of UACR as a Surrogate Endpoint in DKD.

Surrogate Endpoint	Advantages	Limitations
UACR	- Non-invasive and easy to measure with a single morning urine sample. - Strong correlation with 24-hour urine albumin excretion rate. - Established as a reliable marker of albuminuria in DKD. - Reflects early renal damage and is predictive of ESRD and cardiovascular events.	- May not fully capture the severity of renal dysfunction in the absence of albuminuria. - Susceptible to variability based on hydration status or exercise.
Changes in UACR	- Can detect early changes in kidney function, especially in early-stage DKD. - Predictive of adverse renal outcomes such as serum creatinine doubling and the need for renal replacement therapy. - Can be measured at regular intervals for ongoing monitoring of DKD progression.	- Critical thresholds for UACR changes need further validation. - Changes in UACR may be influenced by factors other than kidney function (e.g., infections).
eGFR Slope Combined with UACR Changes	- Provides a more comprehensive assessment of DKD progression by combining two key biomarkers. - eGFR slope reflects gradual kidney function decline, while UACR changes indicate proteinuria severity. - Offers enhanced predictive accuracy for adverse renal outcomes. - Allows for shorter follow-up periods and reduced sample sizes in clinical trials.	- May require longer follow-up periods to detect meaningful changes in both eGFR slope and UACR. - Acute changes in eGFR may interfere with the interpretation of combined outcomes. - Complex analysis may be required for interpretation in certain patient populations.

## 4 Novel biomarkers

As insights into the pathogenesis of diabetic kidney disease (DKD) deepen, a growing array of potential novel biomarkers has been identified. These biomarkers offer significant promise not only for the early diagnosis and prognostication of DKD progression but also as surrogate endpoints for assessing therapeutic efficacy in clinical trials. The biomarkers under investigation reflect diverse pathological processes, including inflammation and oxidative stress. The following section outlines several of these novel biomarkers, highlighting their potential utility as surrogate endpoints in DKD and their prospective applications in clinical trial settings.

### 4.1 Kidney tubular injury biomarkers

Kidney Tubular injury represents a key pathological feature of diabetic kidney disease (DKD). In recent years, urinary biomarkers indicative of tubular damage, such as liver-type fatty acid-binding protein (L-FABP), neutrophil gelatinase-associated lipocalin (NGAL), and kidney injury molecule-1 (KIM-1), have garnered significant research interest. A growing body of evidence has established a strong association between elevated levels of these biomarkers and the progression of DKD, underscoring their potential as early indicators of renal dysfunction.

Kidney injury molecule-1 (KIM-1) is among the most extensively studied biomarkers in diabetic kidney disease (DKD). Elevated urinary levels of KIM-1 can be detected prior to increases in serum creatinine, offering superior sensitivity and specificity compared to UACR, particularly in the early stages of DKD. Moreover, KIM-1 levels have been shown to correlate with declines in estimated glomerular filtration rate (eGFR) (37). In a cohort of 594 diabetic patients with eGFR <60 ml/min/1.73 m², each doubling of KIM-1 concentration was associated with a 1.52-fold increased risk of kidney failure requiring renal replacement therapy (KFRT). Furthermore, KIM-1 remained significantly associated with KFRT events in a Lasso regression model (38). In the CANVAS trial, an increase in baseline KIM-1 levels was strongly linked to the risk of adverse renal outcomes, and treatment with canagliflozin resulted in a 26.7% reduction in KIM-1 levels compared to placebo (39). These findings underscore KIM-1’s potential as not only a reliable marker of tubular injury but also a useful tool for evaluating the efficacy of therapeutic interventions. As such, KIM-1 holds promise as a valuable surrogate endpoint for DKD in clinical trials.

Other tubular injury biomarkers, including neutrophil gelatinase-associated lipocalin (NGAL) and liver-type fatty acid-binding protein (L-FABP), also demonstrate considerable potential in the study of diabetic kidney disease (DKD). Evidence suggests that NGAL plays a pivotal role in the progression of DKD (40). Compared to diabetic individuals with normal proteinuria, both serum NGAL (sNGAL) and urinary NGAL (uNGAL) levels are significantly elevated in DKD patients with microalbuminuria or macroalbuminuria, particularly during the early stages of the disease. Furthermore, both sNGAL and uNGAL exhibit a positive correlation with UACR, further highlighting their relevance as biomarkers for early-stage DKD (41).

Liver-type fatty acid-binding protein (L-FABP) is another prominent biomarker of tubular injury, playing a significant role in the progression of diabetic kidney disease (DKD). Primarily synthesized in the cytoplasm of proximal tubular cells, L-FABP is involved in the metabolism of long-chain fatty acids. An 18-year longitudinal study demonstrated that elevated urinary levels of L-FABP are predictive of DKD progression (42). In a cohort of 227 patients with type 2 diabetes, urinary L-FABP levels were found to be strongly correlated with estimated glomerular filtration rate (eGFR) and UACR, and served as an independent predictor of eGFR decline. Specifically, when urinary L-FABP levels exceeded 6.5 μg/g creatinine, the risk of renal function decline was markedly increased in DKD patients. Further research has shown that treatment with sodium-glucose cotransporter-2 (SGLT-2) inhibitors significantly reduced urinary L-FABP levels, underscoring its potential as a therapeutic target (43). Consequently, both urinary L-FABP and neutrophil gelatinase-associated lipocalin (NGAL) show considerable promise as surrogate endpoints for DKD in clinical settings. Despite these advances, the use of tubular biomarkers as surrogate endpoints in clinical trials remains limited. Key challenges include the lack of assay standardization—variations in sample processing and detection platforms hamper reproducibility and cross-study comparisons. Furthermore, robust validation in large, multicenter cohorts is still underway. To date, no novel tubular injury biomarker has been formally established as a surrogate endpoint in DKD trials.

### 4.2 Inflammatory biomarkers

Metabolism disorders commonly accompany the progression of diabetic kidney disease (DKD) (44, 45). Although some studies have identified associations between metabolic biomarkers, such as lipid biomarkers or bilirubin levels and DKD progression (46–48), these factors have not yet been validated as surrogate endpoints. In contrast, inflammation plays a pivotal role in the pathogenesis of DKD. Numerous studies have demonstrated that inflammatory mediators—including tumor necrosis factor-alpha (TNF-α), monocyte chemoattractant protein-1 (MCP-1), and various interleukins—are significantly elevated in the renal tissue of individuals with DKD (49, 50). These inflammatory biomarkers not only show strong potential in predicting disease progression, but also offer utility in assessing treatment response.

Tumor necrosis factor-alpha (TNF-α) is a pleiotropic cytokine that plays a pivotal role in mediating apoptosis, inflammatory responses, and immune activation. Studies have shown that TNF-α levels are significantly elevated in the renal tissue, serum, and urine of patients with diabetic kidney disease (DKD). These elevations correlate strongly with increased proteinuria and the deterioration of renal function (51). Beyond its role in fostering renal inflammation, TNF-α further exacerbates oxidative stress and apoptotic processes, thereby accelerating the progression of DKD. The tumor necrosis factor (TNF) receptors TNFR1 and TNFR2 are the principal mediators of TNF-α signaling, exhibiting greater stability than TNF-α itself, which makes them promising candidates as surrogate endpoints. A six-year clinical trial investigating diabetic patients revealed a significant association between elevated levels of both TNFR1 and TNFR2 and the risk of adverse renal outcomes in diabetic kidney disease (DKD) (52). Notably, TNFR2 demonstrated a stronger correlation with the progression of DKD compared to TNFR1 (50), and is considered the key receptor influencing the decline in glomerular filtration rate (eGFR) in DKD patients (53). Furthermore, in the CANVAS study, early reductions in both TNFR1 and TNFR2 during treatment with the sodium-glucose cotransporter-2 (SGLT2) inhibitor canagliflozin were associated with a reduced risk of DKD progression (39). These findings underscore the potential of TNFR1 and TNFR2, particularly TNFR2, as valuable surrogate endpoints for evaluating therapeutic efficacy in DKD.

Similarly, MCP-1 is a crucial chemokine involved in the recruitment of monocytes and other inflammatory cells to renal tissue, contributing to renal interstitial inflammation and fibrosis. Elevated MCP-1 levels have been detected in the urine and serum of DKD patients, where they correlate with worsening proteinuria and declining renal function (49). Like TNF-α, MCP-1 amplifies the inflammatory milieu within the kidney, further perpetuating damage and promoting disease progression. Given its pathophysiological relevance and measurable presence in biological fluids, MCP-1 is also being explored as a potential biomarker and surrogate endpoint in DKD. In a study involving 185 patients with type 2 diabetes, the urinary MCP-1-to-creatinine ratio (MCP-1/Cr) was significantly associated with both estimated glomerular filtration rate (eGFR; p = 0.023) and albuminuria (p < 0.001), with levels rising in parallel with increasing kidney damage, demonstrating a clear dose–response relationship (54). Furthermore, in the VA NEPHRON-D study of 1,135 patients with diabetes followed over a median of 2.2 years, those in the highest quartile of urinary MCP-1 had a 2.18-fold increased risk of kidney function decline compared to those in the lowest quartile. A twofold increase in baseline MCP-1 levels was also associated with a 10–40% higher risk of all-cause mortality (55). These findings underscore the potential utility of MCP-1 as a prognostic biomarker and surrogate endpoint in the evaluation of DKD progression.

## 5 Omics-related indicators

In recent years, capillary electrophoresis-mass spectrometry (CE-MS)-based urine proteomics and liquid chromatography-mass spectrometry (LC-MS)-driven targeted metabolomics have emerged as the most promising omics technologies for diabetic kidney disease (DKD) research. These platforms enable high-resolution quantification of disease-specific molecular signatures, offering dynamic insights into early progression and treatment response that complement conventional endpoints like urinary albumin-to-creatinine ratio (UACR) and estimated glomerular filtration rate (eGFR).

Urine proteomics, particularly due to its non-invasive nature and ability to reflect renal function, has emerged as a critical tool in diabetic kidney disease (DKD) research. This approach involves analyzing protein fragments in urine, which serve as key biomarkers for detecting the onset and progression of kidney disease. The urine peptide classifier CKD-273, developed using capillary electrophoresis-mass spectrometry (CE-MS), represents the most extensively studied and validated proteomics classifier to date (56). A prospective randomized controlled trial demonstrated that among 216 high-risk type 2 diabetes patients, 28% progressed to microalbuminuria when classified as high-risk by CKD-273, compared to only 9% in 1,559 low-risk participants (hazard ratio [HR] 2.48, 95% CI 1.80–3.42; p<0.0001). Additionally, 26% of high-risk participants developed impaired kidney function (eGFR <60 mL/min/1.73 m²), while only 8% of low-risk participants did so (HR 3.50, 95% CI 2.50–4.90; p<0.0001), confirming the significant correlation between a high-risk CKD-273 score and the risk of early DKD progression (57).

Targeted liquid chromatography–mass spectrometry (LC–MS) profiling has revealed key mechanistic biomarkers for diabetic kidney disease (DKD), including 3-hydroxyisobutyric acid (3-HIBA), a marker of mitochondrial dysfunction, and branched-chain amino acids (BCAAs) such as leucine and valine, which are associated with insulin resistance (53). In the CRIC prospective cohort study involving 1,001 patients with diabetes followed for a median of 8 years, each standard deviation increase in urinary 3-HIBA was associated with a significantly higher risk of kidney failure requiring replacement therapy (HR = 2.34, 95% CI: 1.51–3.62), and with a faster annual decline in estimated glomerular filtration rate (eGFR) (58). Additionally, a recent study of 2,670 diabetic kidney disease (DKD) patients identified seven urinary metabolites, including leucine, valine, and isoleucine, which were closely correlated with the progression of DKD (59). Notably, elevations in BCAAs and 3-HIBA occurred during normoalbuminuria, providing a temporal advantage over conventional albuminuria-based markers. These metabolic alterations offer a critical window for early risk stratification and personalized intervention in DKD.

Despite significant advances in urinary proteomics and metabolomics for diabetic kidney disease (DKD), clinical translation remains limited by several critical challenges. Chief among these are the inherent complexity of omics data and the lack of methodological standardization across studies. Three major barriers hinder progress toward clinical application. First, technical variability is substantial, differences in urine collection protocols (e.g., fasting vs. random sampling, use of protease inhibitors) markedly affect the stability of peptides and metabolites. Second, biological heterogeneity complicates interpretation. Comorbidities such as obesity can elevate branched-chain amino acids, necessitating stratified analyses to isolate DKD-specific signals. Third, external validation is lacking. Most studies have focused on European populations—for example, 89% of participants in the PRIORITY trial were White—leaving DKD phenotypes in Asian and African populations underexplored (52). To overcome these limitations, future efforts should prioritize the optimization of analytical platforms, the establishment of standardized data protocols, and the validation of candidate biomarkers in large, ethnically diverse cohorts.

## 6 Conclusions

**Figure 1:**
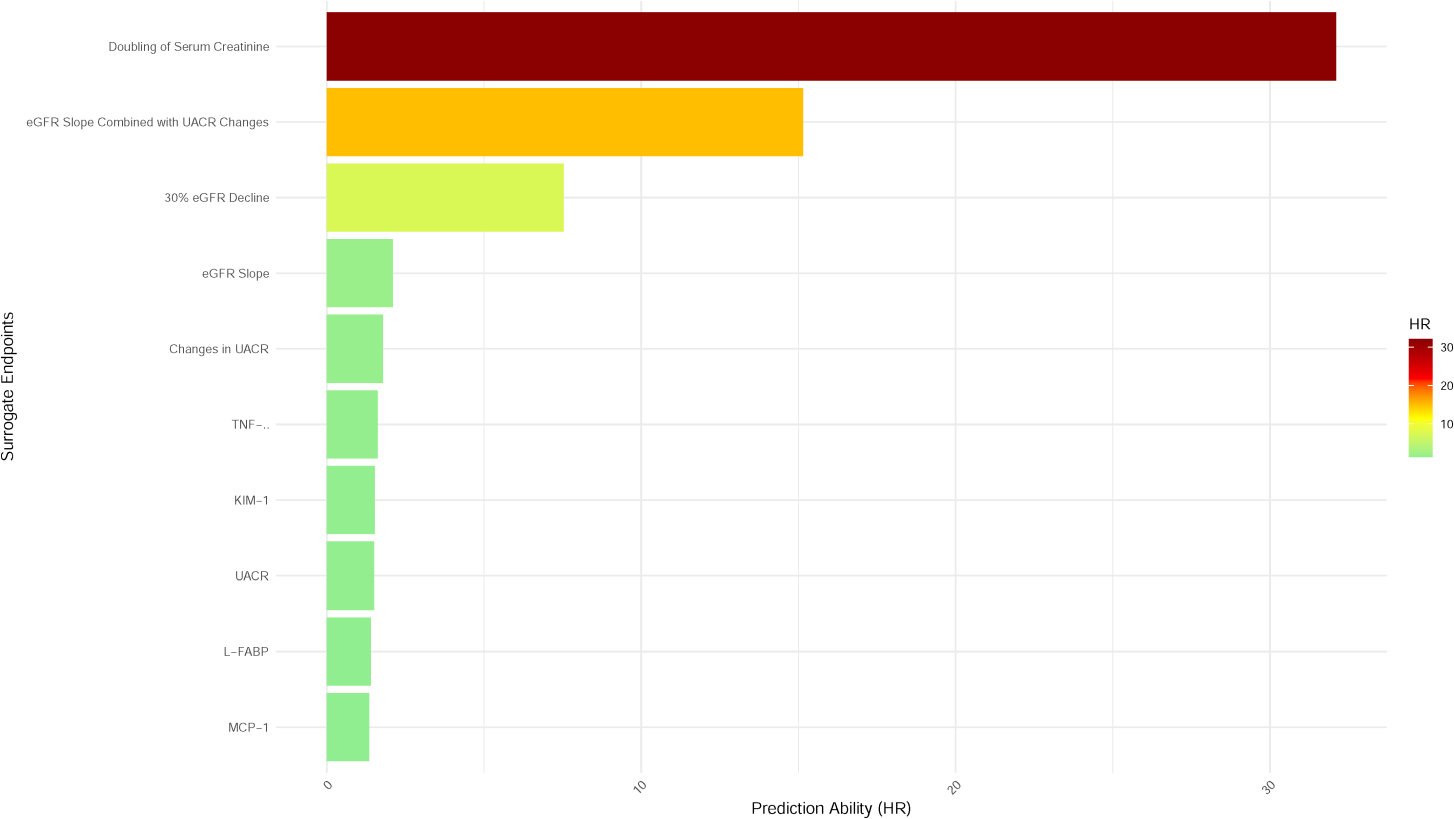
Predictive performance of surrogate endpoints for ESRD in diabetic kidney disease. Bar plot illustrating the prediction ability (hazard ratio, HR) of various surrogate endpoints for progression to ESRD in patients with diabetic kidney disease (DKD). Endpoints are ranked by descending HR, reflecting their predictive strength. The doubling of serum creatinine demonstrated the highest predictive value (HR > 30), followed by the eGFR slope combined with changes in UACR. Biomarkers such as interleukin-1β (IL-1β), kidney injury molecule-1 (KIM-1), liver-type fatty acid-binding protein (L-FABP), and monocyte chemoattractant protein-1 (MCP-1) exhibited lower predictive capacities. Color gradients represent HR magnitude, with darker hues indicating stronger predictive performance.

Diabetic kidney disease (DKD) represents a major complication of diabetes, often progressing to end-stage renal disease (ESRD), a condition that severely compromises patients’ quality of life and imposes significant healthcare costs. Early identification and intervention in DKD are therefore critical. The use of surrogate endpoints in clinical trials has become an essential strategy for evaluating the efficacy of novel therapies, enabling a reduction in trial duration and associated costs. Despite the widespread application of various surrogate endpoints, their predictive capacity for DKD progression remains heterogeneous. Among these, serum creatinine doubling is regarded as the most robust predictor of DKD progression, while the predictive potential of emerging biomarkers typically ranges between 1 and 2. The specific predictive values are depicted in **Figure 1**.

Currently, the rate of change in estimated glomerular filtration rate (eGFR) and alterations in the urine albumin-to-creatinine ratio (UACR), whether assessed independently or in combination, serve as reliable surrogate endpoints for monitoring DKD progression. Although novel biomarkers such as KIM-1 and TNFR2 have not yet been established as standalone surrogate endpoints for DKD, their predictive utility may be enhanced when used in conjunction with established markers, such as eGFR slope and UACR change, to improve risk prediction for ESRD. While omics-based biomarkers show promise in DKD research, their clinical applicability requires further validation, particularly through long-term follow-up studies and dynamic monitoring, to confirm their effectiveness.

Clinical trials must account for various confounding factors that can influence the relationship between surrogate endpoints and endpoints. These include baseline clinical variables, the effects of systemic inflammation and metabolic disturbances, the stage of chronic kidney disease (CKD), baseline proteinuria, differences in treatment regimens, and inconsistencies in how proteinuria and albuminuria are measured. Such factors can introduce significant variability in the performance of surrogate endpoints. Future research should prioritize large-scale, multi-ethnic, long-term prospective cohort studies to validate the predictive accuracy of both established and emerging biomarkers. The development of integrated predictive models that combine eGFR slope, changes in urinary albumin-to-creatinine ratio (UACR), and promising biomarkers such as TNFRs and KIM-1 holds great potential to enhance the precision of end-stage renal disease (ESRD) risk stratification. These efforts are essential for the identification of clinically relevant surrogate endpoints and the advancement of precision medicine in diabetic kidney disease.
